# Evaluation of a non‐radioactive rapid test for determination of serum progesterone in South American camelids

**DOI:** 10.1002/vetr.6027

**Published:** 2025-12-04

**Authors:** Lina Längerer, Gerhard Schuler, Philipp Pohlscheid, Henrik Wagner, Kathrin Büttner, Axel Wehrend

**Affiliations:** ^1^ Veterinary Clinic for Reproductive Medicine and Neonatology Justus Liebig University Giessen Giessen Germany; ^2^ Unit of Biomathematics and Data Processing, Faculty of Veterinary Medicine Justus Liebig University Giessen Giessen Germany

**Keywords:** alpaca, endocrinology, llama, oestrus cycle, pregnancy, progesterone, reproduction

## Abstract

**Background:**

Determination of the serum progesterone (P4) concentration is of high importance in reproductive medicine and may be applied for diagnosing pregnancy in South American camelids. P4 measurements in alpacas and llamas are usually performed using immunoassays, which are time consuming and require appropriate expertise of laboratory staff. In this study, the suitability of a commercial automated enzyme‐linked immunofluorescence assay using a mini‐VIDAS device (bioMérieux) for P4 determination in South American camelids was investigated.

**Methods:**

Serum was prepared from the whole blood samples from 116 alpacas and 73 llamas; each sample was divided into two aliquots and stored at –20°C. Comparative P4 measurements were performed using the mini‐VIDAS method and an in‐house radioimmunoassay as the reference standard.

**Results:**

The results showed a strong positive correlation between the results of the two methods (Spearman's rank correlation alpaca: rs = 0.9168 [*p* < 0.05]; llama: rs = 0.9040 [*p* < 0.05]).

**Limitations:**

Although the measuring methods are highly correlated, deviations from the reference method occurred in the high measurement range of mini‐VIDAS.

**Conclusions:**

This study confirmed that the mini‐VIDAS is suitable for determining serum P4 concentration in domestic South American camelids and provides reliable results in less than 1 hour.

## INTRODUCTION

The measurement of sex steroids plays a prominent role in reproductive endocrinology diagnostics and research. Despite the species‐independent structure of steroid hormones, the analytical methods usually established for use in humans or laboratory rodents cannot be used in veterinary medicine without further ado due to interfering factors from the sample material (matrix effects). Matrix effects can mean that a measurement method that works reliably in a certain species or a certain type of sample material is only of limited value or completely unusable in another species or in another kind of substrate. Therefore, appropriate validation of the test method is required before introducing a test as a routine method in an animal species in the laboratory. Due to matrix effects, even reference values published in the literature cannot be used without further ado for the interpretation of data obtained from a different analytical test.^1^

Determining serum progesterone (P4) levels to assess corpus luteum function and indicate pregnancy is very important in different species.^2^, ^3^, ^4^ The llama (*Lama glama*) and alpaca (*Vicugna pacos*) are induced ovulators.^5^ Before mating, the P4 concentration is basal (<2 ng/mL, but generally below 0.1 ng/mL when using sufficiently sensitive assays).^6^ Ovulation and formation of corpora lutea and secretion of P4 occur 44‒48 hours after mating in llamas and 24‒26 hours after mating in alpacas.^7^, ^8^ From day 4 or 5 after mating, P4 concentration in the blood is elevated.^9^, ^10^ Maximum concentrations of 3‒6 ng/mL are reached on days 7‒8. At this time, serum P4 concentrations equal to or higher than 1 ng/mL indicate ovulation and luteal activity but not necessarily pregnancy.^2^ This is followed by a rapid decrease in serum P4 concentration on days 9‒10 after breeding, due to luteolysis.^5^, ^10^ A pregnancy is suspected in llamas on day 21 after breeding when serum P4 concentration is higher than 1 ng/mL.^2^, ^3^ Two P4 measurements should be carried out 12‒14 days apart to diagnose pregnancy in alpacas and llamas. A P4 concentration of more than 2 ng/mL indicates functional corpus luteum tissue and suggests pregnancy. Owing to the high embryonic mortality, some authors recommend that the first P4 measurement should not be performed before 21‒30 days after mating.^11^

P4 levels remained at more than 2 ng/mL during pregnancy.^6^, ^9^, ^12^ Adam et al. observed P4 concentrations between 5 and 12 ng/mL throughout pregnancy with slightly lower concentrations after 4 months of gestation.^13^ Seventy‐two hours prior to parturition, P4 concentration starts to decrease and reaches less than 2 ng/mL between 1 day antepartum and the day of delivery.^6^, ^9^

The mini‐VIDAS progesterone kit is a commercial enzyme‐linked immunofluorescence assay (ELFA) running on an automated platform (mini‐VIDAS; bioMérieux). Initially, developed for measuring P4 in human blood samples, it has been subsequently evaluated for P4 levels assessed in the sera of dogs and horses.^14^, ^15^ The aim of this study was to assess the suitability of the automated, rapid and non‐radioactive ELFA method for measuring P4 concentrations in alpaca and llama serum samples.

## MATERIALS AND METHODS

### Collection and processing of blood samples

In total, blood samples from 116 alpacas and 73 llamas were evaluated. Material originated partly from stored samples of the Veterinary Clinic for Reproductive Medicine and Neonatology at Justus Liebig University Giessen, partly from blood taken for diagnostic investigations, as well as from samples previously collected for progesterone measurements within another project. All owners provided consent for the use of animal data in this study. Sampling was carried out in adult camelids representing different reproductive stages. Venipuncture was performed at the jugular vein and blood was collected into S‐Monovette tubes (Sarstedt AG & Co.) containing clot activator, followed by serum preparation.

### Determination of serum P4 concentration

P4 levels were measured in the first aliquot of each sample using the mini‐VIDAS method.

The determination of serum P4 concentrations using the mini‐VIDAS system (bioMérieux) is based on the principle of an ELFA, a competitive immunoassay that combines antigen–antibody binding with enzymatic fluorescence detection. After venipuncture, blood samples are centrifuged and serum is obtained as the analytical material. In contrast to classical radioimmunoassay (RIA) techniques, no laborious extraction with organic solvents is required, which makes the method more user friendly and less time consuming.^1^ Within the assay cassette, a solid‐phase antibody specifically binds P4. Serum P4 competes with a fluorescently labelled progesterone analogue for these binding sites. After washing steps, the remaining fluorescence signal is recorded and is inversely proportional to the concentration of P4 in the sample.^14^, ^16^

The system allows the simultaneous measurement of up to 12 samples within approximately 45 minutes, with a validated measuring range between 0.25 and 80 ng/mL.^16^ This range covers clinically relevant P4 concentrations in camelids.^2^, ^6^ The mini‐VIDAS has already been successfully validated in several species, including humans, dogs and horses, and shows high comparability with RIA results, although slight proportional differences may occur due to assay‐specific characteristics.^15^, ^17^

In contrast to the established RIA method described by Hoffmann et al.,^18^ which relies on tritium‐labelled tracers and therefore involves handling of radioactive material, the mini‐VIDAS assay avoids such safety and regulatory challenges. The measurements were performed according to the manufacturer's instructions.

For reference purposes, the second aliquot was analysed by a validated in‐house RIA according to Hoffmann et al.^18^ The application of this assay has been described in various domestic animal species.^15^, ^17^ Prior to RIA, 0.1 mL serum was extracted twice with hexane in order to reduce matrix effects. After evaporation, the pooled extracts were reconstituted in phosphate buffer (pH 7.2) supplemented with 0.1% bovine serum albumin. The antiserum characteristics were as follows: cross‐reactivity with P4 100%, pregnenolone 0.69%, 17α‐hydroxyprogesterone 0.49%, testosterone 0.37% and less than 0.01% for androstenedione, estradiol‐17β, estrone and cortisol.^17^ Quantification was performed using a tritium‐labelled P4 tracer ([1,2,6,7‐3H] P4; PerkinElmer). Separation of bound and free hormone fractions was achieved by adsorption of free steroids with activated charcoal. All samples were assayed in duplicate. The intra‐ and inter‐assay coefficients of variation were 8.8% and 8.9%.^19^ The standard radioimmunological measurements ranged from 0.1 to 8.0 ng/mL. In the case of measured values above the standard curve, re‐testing was performed using a smaller sample volume.

### Data analysis

For method comparison, Passing‒Bablok regression and Spearman's rank correlation were applied. The Passing‒Bablok regression was also subjected to a CUSUM test for linearity. The calculations were done using the software SAS 9.4, 2013 (SAS Institute), and the significance level was set to *α* = 0.05.

For values below the detection limit of both analytical methods (RIA <0.1 ng/mL; mini‐VIDAS <0.25 ng/mL), the detection limit was defined as the P4 value (RIA = 0.1 ng/mL; mini‐VIDAS = 0.25 ng/mL). Since the clinically relevant measuring range is approximately 1‒4 ng/mL, a further calculation was carried out with the samples whose measured values from the RIA were in this measuring range, including samples of 43 llamas and 74 alpacas. The aim was to obtain a regression line with the clinically relevant measurement range.

## RESULTS

Across both species, the two measurement methods had a strong positive correlation. The slope of the regression line showed that the 95% confidence interval did not include 1, indicating a proportional deviation between methods. The 95% confidence interval for the intercept contains 0, so there was no systematic difference between the two methods. The graph shows that the methods are linearly related, and a CUSUM test for linearity was performed. Without calculating values below 1 ng/mL, there was a high correlation between the measurement methods. The CUSUM test for linearity was not significant; therefore, it can be assumed that the measurement methods are linearly related.

### Alpacas

With all measurement values included in the calculation, the Spearman's rank test showed a correlation of rs = 0.93412 (*p* < 0.05). The equation used for the Passing‒Bablok regression was RIA = 0.049 + 0.65 × mini‐VIDAS (Figure 1).

**FIGURE 1:**
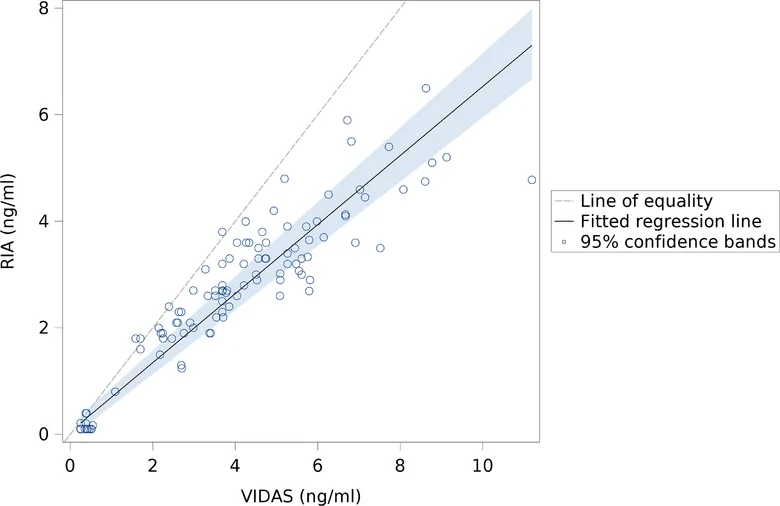
Comparison of the two progesterone measurement methods (ng/mL) when applied to the serum of alpacas. The Passing‒Bablok regression line of all values with 95% confidence intervals is shown. Dotted line: line of equality

When only concentrations between 1 and 4 ng/mL were used, the equation for Passing‒Bablok regression was RIA = 0.748 + 0.51 × mini‐VIDAS (Spearman rank correlation rs = 0.80520 [*p* < 0.05]).

### Llamas

The Spearman rank test showed a correlation of rs = 0.91763 (*p* < 0.05) for all P4 concentrations included in the calculation. The equation used for the Passing‒Bablok regression was RIA = 0.0844 + 0.68 × mini‐VIDAS (Figure 2) when all measurement values were included in the calculation.

**FIGURE 2:**
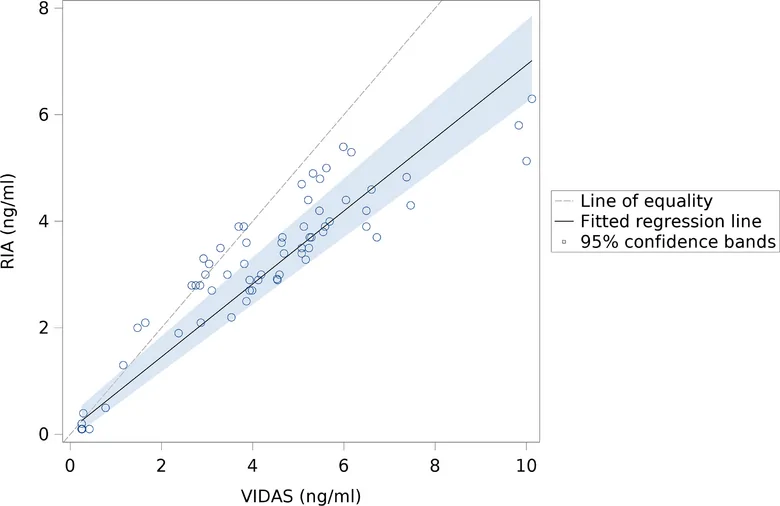
Comparison of the two progesterone measurement methods (ng/mL) when applied to the serum of llamas. The Passing‒Bablok regression line of all values with 95% confidence intervals is shown. Dotted line: line of equality

With a measurement range of 1‒4 ng/mL, the equation of the Passing‒Bablok regression equation was RIA = 1.19169 + 0.47506 × mini‐VIDAS, with a Spearman rank correlation of rs = 0.72628 (*p* < 0.05).

## DISCUSSION

The use of a mini‐VIDAS device to measure P4 concentrations in alpaca and llama serum samples was evaluated in this study. As previously found for other species,^14^, ^15^ the mini‐VIDAS overestimates P4 levels compared to RIA, but there is still a strong positive correlation between the measurement methods. Part of the overestimation by the mini‐VIDAS can be explained by the fact that this method measures directly, while RIA is preceded by sample extraction, which is associated with a loss of the analyte of approximately 5%‒10%. The fact that the mini‐VIDAS overestimation was higher than the extraction loss in the RIA indicates that mini‐VIDAS is sensitive to species‐specific matrix effects in the tested domestic mammals. Despite considerable overestimation due to the pronounced positive correlation with the reference method, mini‐VIDAS can be considered a valuable method for precise and rapid P4 determination in a clinical setting in the target species tested here. Previous studies calculated the proportionality factors for multiple species to avoid determining method‐specific reference values.^14^, ^15^, ^17^ This allows the use of existing reference values by multiplying the results of the mini‐VIDAS with a constant factor. In the animal species examined in this study, multiplying the results from the mini‐VIDAS alone by a proportionality factor led to results with unsatisfactory consistency with the reference method, as the equation of the regression line shows a considerable axis shift. Therefore, the values measured with the mini‐VIDAS should be used in the following linear equations to determine the P4 concentrations:

Alpacas:RIA=0.75+0.51×mini−VIDAS

Llamas:RIA=1.2+0.48×mini−VIDAS

The differences between the equations indicate species‐specific variation, which is presumably attributable to matrix effects.^1^ This has already been demonstrated in other animal species.^14^, ^15^, ^17^

Regular control measurements should be performed because commercial immunoassay kits intended for humans are occasionally subject to manufacturer changes that may affect their use in veterinary medicine.^20^ The applicability across different animal species represents an advantage for clinical use, as does the short analysis time of 45 minutes and the fact that the method does not require the use of a radioactive tracer.^21^

As already observed for other species, the consistency of the measurement methods decreased in the higher measurement range in this study, which is evident from the 95% confidence interval. One possible cause is the poor linearity of one or both measurement methods. However, the linearity of RIA was confirmed in the defined measurement range of 0.1 and 8 ng/mL. For high P4 concentrations in the upper measurement range or above, the measurements were repeated with a reduced sample volume to rule out a significant linearity problem for RIA. The lower agreement in higher P4 values could also be caused by different cross‐reactivities of the antisera used with P4 metabolites, which can be detected both in the luteal phase in cyclic animals^22^ and in pregnancy.^23^ An evaluation including a larger cohort of animals, with reproductive and gestational stages being systematically assessed and documented, may contribute to a better understanding of this issue.

Mini‐VIDAS is suitable for serum progesterone determination in alpacas and llamas, if the corresponding linear equation is used to calculate the result based on the measured value.

## AUTHOR CONTRIBUTIONS

*Data curation (lead), methodology, writing—original draft (lead) and writing—review and editing (lead)*: Lina Längerer. *Conceptualisation (lead), methodology (lead) and writing—review and editing (lead)*: Gerhard Schuler. *Data curation (supporting) and writing—review and editing (supporting)*: Philipp Pohlscheid. *Data curation (supporting) and writing—review and editing (supporting)*: Henrik Wagner. *Conceptualisation (lead), methodology (lead), supervision (lead) and writing—review and editing (lead)*: Axel Wehrend. *Data curation (lead), formal analysis (lead) and visualisation*: Kathrin Büttner.

## CONFLICT OF INTEREST STATEMENT

None of the authors have financial or personal relationships that could influence or affect the content of this work.

## FUNDING INFORMATION

No funding has been received.

## ETHICS STATEMENT

Ethical approval was granted by the Regional Council of Giessen (KTV 7‐2017; V 54‐19 c 18‐V/9001).

## Data Availability

The data that support the findings of this study are available from the corresponding author upon reasonable request.
